# Factorial invariance of the Patient Health Questionnaire and Generalized Anxiety Disorder Questionnaire

**DOI:** 10.1111/bjc.12028

**Published:** 2013-08-20

**Authors:** Travis A Ryan, Alastair Bailey, Pasco Fearon, John King

**Affiliations:** 1 Research Department of Clinical, Educational and Health Psychology, University College LondonUK; 2 Camden Psychological Therapies ServiceLondon, UK

**Keywords:** IAPT, PHQ-9, GAD-7, Confirmatory factor analysis, Factorial invariance

## Abstract

**Objectives:**

The UK's Improving Access to Psychological Therapies (IAPT) programme uses the Patient Health Questionnaire Depression Scale (PHQ-9; Kroenke, Spitzer, & Williams, 2001, *J. Gen. Intern. Med*., 16, 606) and Generalized Anxiety Disorder Scale (GAD-7; Spitzer *et al*., 2006, *Arch. Intern. Med*., 166, 1092) to assess patients' symptoms of depression and anxiety respectively. Data are typically collected via telephone or face-to-face; however, no study has statistically investigated whether the questionnaires' items operate equivalently across these modes of data collection. This study aimed to address this omission.

**Methods & Results:**

Questionnaire data from patients registered with an IAPT service in London (*N* = 23,672) were examined. Confirmatory factor analyses suggested that unidimensional factor structures adequately matched observed face-to-face and telephone data for the PHQ-9 and GAD-7. Invariance analyses revealed that while the PHQ-9 had equivalent factor loadings and latent means across data collection methods, the GAD-7 had equivalent factor loadings but unequal latent means. In support of the scales' convergent validity, positive associations between scores on the PHQ-9 and GAD-7 emerged.

**Conclusions:**

With the exception of the GAD-7's latent means, the questionnaires' factor loadings and latent means were equivalent. This suggests that clinicians may meaningfully compare PHQ-9 data collected face-to-face and by telephone; however, such comparisons with the GAD-7 should be done with caution.

**Practitioner points:**

- The PHQ-9 and GAD-7's factor loadings were equivalent across data collection methods.
- Only the PHQ-9's latent means were equivalent across data collection methods.
- Clinicians may be confident collecting PHQ-9 data by telephone and face-to-face and, then, comparing such data.
- Caution is recommended when determining clinical effectiveness using telephone and face-to-face GAD-7 data.
- More psychometric research is warranted.

In 2007, the UK government launched an initiative for Improving Access to Psychological Therapies (IAPT) for depression and anxiety disorders within the English National Health Service. It aimed to provide evidence-based psychological therapies recommended by the National Institute for Clinical Excellence using a stepped-care approach to the delivery of psychological therapy. Employees of IAPT are instructed to use the 9-item Patient Health Questionnaire Depression Scale (PHQ-9; Kroenke, Spitzer, & Williams, 2001) and the 7-item Generalized Anxiety Disorder scale (GAD-7; Spitzer, Kroenke, Williams, & Löwe, 2006) in every session to assess patients' depression and anxiety, respectively. Sessional outcome data collection might improve patient communication, clinical decision-making, case supervision, and interprofessional communication, and permit end-of-treatment scores when therapy unexpectedly ends (Department of Health, 2011). While most data are collected face-to-face, some are received by telephone. Assessment, post-treatment, and follow-up scores on the PHQ-9 and GAD-7 are investigated to determine clinical outcomes (Clark *et al*., 2009). Therefore, to determine the effectiveness of their psychological interventions, clinicians sometimes have to compare data that are collected in different ways.

Data collection methods may differentially affect data, with the absence of visual support in telephone data collection likely to increase the cognitive burden on participants and make the establishment of a positive rapport between the patient and clinician difficult to achieve (Bowling, 2005; Evans, Kessler, Lewis, Peters, & Sharp, 2004). Research has documented differences between data collected via telephone and face-to-face (Bowling, 2005; Evans *et al*., 2004), with Evans *et al*. finding that older English primary care patients reported greater depressive symptomatology by telephone, and most patients indicated a preference for face-to-face data collection.

Despite this, no research has investigated if the PHQ-9 and GAD-7's factor structures (the intercorrelations among the questionnaire items that are indicators of their underlying latent constructs) are invariant (i.e., equivalent) across data collection methods, such as telephone or face-to-face. That is, factorial invariance of the PHQ-9 or GAD-7 has not been established. The establishment of factorial invariance, an empirical issue of fundamental importance (Meredith & Teresi, 2006), is required if meaningful comparisons of questionnaire data across groups are to be made (Milfont & Fischer, 2010). There are different types of factorial invariance (Byrne & Stewart, 2006; Meredith & Teresi, 2006). The first is factor loading invariance (i.e., the equivalence of factor loadings [the relationships between items and the latent variable] across groups). If factor loadings are not invariant, the questionnaire items are not assessing the latent variable in the same way across groups; therefore, people cannot meaningfully compare persons' scores on the items or their latent variable across groups (Meredith & Teresi, 2006).

However, factor loading invariance alone is insufficient to ensure meaningful group comparisons because it does not permit comparisons of factor means (Meredith & Teresi, 2006). A more restrictive test of factorial invariance examines the equality of latent means. The establishment of this form of factorial invariance, known as strong invariance, allows for comparisons of latent construct means across groups. As the means of latent constructs are unobservable, they are derived from their indicator variables (i.e., questionnaire items). This test of invariance examines the equivalence of means related to each latent construct or, in other words, it determines if latent means are different (Byrne, 2001). The last and most stringent type of factorial invariance, strict invariance, requires invariant item residual variances across groups (Meredith & Teresi, 2006); however, this kind of invariance is unreasonable, not recommended, and the least important (Byrne & van de Vijver, 2010).

Given this, the current research investigated the factorial invariance of the PHQ-9 and GAD-7, examining whether patients' scores when they first contacted their IAPT service differed according to their collection method (i.e., face-to-face vs. telephone). Specifically, the invariance of factor loadings and item means will be investigated. A secondary aim was to test the convergent validity of the PHQ-9 and GAD-7. As Spitzer *et al*. (2006) documented a positive correlation between the GAD-7 and the PHQ-8 (Kroenke *et al*., 2009), a positive association was likewise anticipated between the GAD-7 and the PHQ-9.

## Method

### Participants

Participants were 23,672 IAPT patients living in a borough of London. They were aged 16–112 (*M* = 40.02; *SD* = 13.68; 0.2% did not report their age), of whom 64.5% were female and 33.4% were male. Others did not specify their gender (1.8%) and there were missing data (0.2%). Participants' self-reported ethnicities were as follows: White British (43.4%); other White background (16.5%); White Irish (3.5%); Black African British (2.4%); Black Caribbean British (2.4%); other ethnic groups (2.3%); Bangladeshi British (2.0%); other mixed background (1.7%); other Asian background (1.6%); Indian British (1.2%); White and Black Caribbean British (1.1%); other Black British (1.0%); Chinese (0.7%); White and Asian (0.7%); White and Black African (0.6%); Pakistani British (0.5%); Black Somali British (0.3%); and White Turkish or Turkish Cypriot (0.2%). Others did not want to report their ethnicity (5.1%) and some data were missing (12.1%). The majority of patients provided data face-to-face (78.2%; *n* = 18,501), with a minority giving responses by telephone (21.5%; *n* = 5,084). Small proportions of the sample provided their data via another means (0.2%), and other data were missing (0.2%).

### Measures

#### Patient Health Questionnaire (PHQ-9; Kroenke *et al*., 2001)

The PHQ-9 is a 9-item self-report measure of depression severity (Kroenke *et al*., 2001). The PHQ-9 asks how often participants have been bothered by problems in the past 2 weeks. Then, nine symptoms are listed (e.g., little interest or pleasure in doing things). Response options are as follows: Not at all; Several days; More than half the days; and Nearly every day. Scores may range from 0 to 27, with higher scores indicating greater depression. Kroenke *et al*. examined the PHQ-9's psychometric properties among patients from the United States. Favourable Cronbach's alphas (e.g., α =. 89) suggested that the PHQ-9 yielded internally consistent scores, and several strands of evidence attested to the scale's validity (e.g., as anticipated, scores on the PHQ-9 positively associated with functional impairment). Factor analyses have suggested the PHQ-9's unidimensionality (Cameron, Crawford, Lawton, & Reid, 2008; Dum, Pickren, Sobell, & Sobell, 2008; Huang, Chung, Kroenke, Delucchi, & Spitzer, 2006).

#### Generalized Anxiety Disorder Questionnaire (GAD-7; Spitzer *et al*., 2006)

The GAD-7 is a 7-item self-report scale recommended for screening for generalized anxiety disorder and evaluating its severity (Spitzer *et al*., 2006). Although it was developed to assess generalized anxiety disorder, Kroenke, Spitzer, Williams, Monahan, and Löwe (2007) found that the GAD-7 has good sensitivity and specificity for other anxiety disorders (e.g., panic disorder) too. Participants are asked the degree to which seven symptoms (e.g., ‘feeling nervous, anxious or on edge’) have bothered them during the preceding 2 weeks. The response options are as follows: Not at all; Several days; More than half the days; and Nearly every day. Higher scores denote greater generalized anxiety (the possible range is 0–21). Research has attested to the scale's psychometric soundness (Spitzer *et al*., 2006; Kroenke *et al*., 2007). For example, Spitzer *et al*. (2006) examined its psychometric properties among a sample of patients in the United States. The scale's reliability was supported by a Cronbach's alpha of. 92 and a test–retest reliability coefficient of. 83, and several indicants of validity were reported (e.g., as expected, scores on the GAD-7 positively associated with scores on other anxiety questionnaires). Spitzer *et al*. also carried out a principal components analysis on the GAD-7 and a measure of depression, with results indicating that the GAD-7 was unidimensional. Other research has similarly demonstrated the GAD-7's one-dimensional factor structure (Dear *et al*., 2011; Löwe, Decker *et al*., 2008).

### Procedure

Data collected between 20 August 2008 and 14 June 2012 were imported from the patient case management information system (PC-MIS). An SPSS data set with data on patients' first contact with the IAPT service was then created.

### Statistical analyses

A missing values analysis was conducted in accord with best practice guidelines (Jeličić, Phelps, & Lerner, 2009; Schlomer, Bauman, & Card, 2010). There were missing values on all 20 variables (i.e., nine PHQ-9 items, seven GAD-7 items, age, gender, mode of data collection, and ethnicity); 17.47% of participants had missing values; and 5.01% of values were missing. Missing values ranged from a low of 0.2% for mode of data collection to a high of 12.1% for ethnicity. Item 5 had the most missing values for the GAD-7 (5.7%), and item 8 had the most missing values for the PHQ-9 (5.5%). Missing values were not missing completely at random, χ^2^(1,242) = 1,390.64, *p* <. 01, and the data set was non-monotone. Therefore, as recommended by researchers (Jeličić *et al*., 2009; Schlomer *et al*., 2010), multiple imputation was used.

The fully conditional specification imputation method was used with five imputations; the maximum number of parameters in the imputation model was one thousand; and constraints were imposed on the age variable (i.e., in accordance with original data, the minimum age was 16 and the maximum age was 112). Descriptive statistics showed negligible differences between the multiply imputed data sets and the original data (e.g., *M*s and Cronbach's alpha values only differed at the second decimal point). Similarly, analyses on each data set produced results that were almost identical.

After computing descriptive statistics and reliability estimates, confirmatory factor analysis (CFA) was conducted on each questionnaire using IBM SPSS AMOS 19 (Amos Development Corporation, Crawfordville, FL, USA). Confirmatory factor analyses were carried out on each multiply imputed data set and results were pooled (Enders, 2010; Rubin, 1987). For CFA, Hoyle (2000) recommends using fit statistics that possess different computational logic. Thus, absolute fit was assessed using the standardized root mean square residual (SRMR) and the root mean square error of approximation (RMSEA), and comparative fit was examined using Bentler's Comparative Fit Index (CFI). Suggested guidelines for these indices are as follows: SRMR close to. 08 (Hu & Bentler, 1999); RMSEA close to. 06 (Hu & Bentler, 1999); and CFI close to. 95 (Hu & Bentler, 1999). The Akaike information criterion (AIC) was used to compare the relative fit of models, with lower AIC values indicating superior model fit. As recommended by Burnham and Anderson (2002), delta AIC (higher AIC − lower AIC) was used to assess empirical support for inferior models (i.e., models with higher AIC values) relative to their superior counterparts (i.e., models with lower AIC values). Substantial support for the inferior model is suggested by values of 0–2, considerably less support is suggested by delta AICs of 4–7, and values of 10 or more should be interpreted as signalling essentially no support for the model with the higher AIC (Burnham & Anderson, 2002). Modification indices also were inspected to assess the extent to which the hypothesized model was appropriately described (Byrne, 2001).

Next, IBM SPSS AMOS 19 was used to test the questionnaires' factor loading invariance using Byrne's (2001) guidelines. Even if models are similarly specified for face-to-face and telephone data, this does not guarantee the equivalence of item measurements. The link between an item and the latent variable may differ across modes of data collection. Therefore, this should be statistically tested (Byrne, 2001). First, the factor structure of each questionnaire was confirmatory factor analysed, estimating parameters for face-to-face and telephone data at the same time. The simultaneously estimated models' fit provided the baseline value against which subsequently specified models were compared. Then, the baseline models were compared with fully constrained models. Constraints were placed on all parameters (i.e., they were specified as invariant), including error covariances when conceptually justified, to test invariance across groups. Chi-square values then were compared, with statistically significant differences suggesting non-invariance (Byrne, 2001). However, as chi-square difference values are sensitive to sample size, the following criteria were followed to determine measurement invariance: ‘(1) the multigroup model exhibits an adequate fit to the data and (2) the ΔCFI values between models is negligible' (Byrne, Stewart, Kennard, & Lee, 2007, p. 303). Cheung and Rensvold (2002) stated that the change in CFI values (i.e., CFI_constrained_ − CFI_unconstrained_) provides the best information for determining measurement invariance with negative ΔCFI values lower than −.01 indicating a lack of invariance (Dimitrov, 2010).

The invariance of each questionnaire's latent mean structures across the two modes of data collected was then tested. The baseline models included error covariances as appropriate, and factor loadings (except one loading fixed to 1) were constrained to be equal across groups. Means of the error terms were constrained to zero; intercepts for observed variables were constrained equal across groups; and the factor means for face-to-face data were freely estimated, whereas the factor means for telephone data were constrained to zero (i.e., the latter were the reference groups). To compare the models, critical ratio values for latent mean estimates were inspected, along with the goodness of fit between the hypothesized models and the multigroup data (Byrne, 2001).

Lastly, Pearson's *r* correlations tested the convergent validity of the PHQ-9 and GAD-7. Results across the multiply imputed data sets were pooled (Enders, 2010; Rubin, 1987).

## Results

Descriptive and reliability statistics are given in Table 1. Regardless of data collection mode, participants' general anxiety and depression typically were in the moderate range.

**Table 1:** Descriptive and reliability statistics

Measure	Mean	*SD*	Alpha	95% CI
PHQ-9 (face-to-face)	14.13	6.86	.88	.88–.89
PHQ-9 (telephone)	14.25	6.16	.83	.82–.84
GAD-7 (face-to-face)	12.42	5.64	.89	.88–.89
GAD-7 (telephone)	12.98	4.97	.82	.81–.83

*Note*. PHQ-9 = Patient Health Questionnaire (Kroenke *et al*., 2001); GAD-7 = Generalized Anxiety Disorder Scale (Spitzer *et al*., 2006).

### Confirmatory factor analyses of the PHQ-9

Face-to-face data emerged as multivariate non-normal (Mardia's coefficient = 7.88), with several variables exceeding the critical value for Mahalanobis distance (i.e., 27.88 for nine dependent variables). Given problems associated with maximum likelihood (ML) estimation under non-normal conditions, analyses were carried out with and without bootstrapping, which is not based on the assumption of normal distribution and, relative to ML estimation, provides standard error estimates that are less biased (Byrne, 2001). However, as comparable results emerged, we report the output of the ML estimation.^1^

Fit indices for a unidimensional PHQ-9 were good and the largest modification index indicated overlap in content between items 7 and 8, which makes sense because concentration and restlessness may be perceived as closely related. When their error variances were correlated, improved fit indices emerged. The second largest modification index suggested that there was shared content between items 3 and 4, which relate to sleep and tiredness respectively. When their error variances were allowed to correlate, fit indices improved again. Table 2 contains the fit indices for the above models.

**Table 2:** Confirmatory factor analysis for PHQ-9 by mode of administration

Mode of administration	*df*	Chi-square^a^	SRMR	RMSEA (90% CI)	CFI	AIC	Delta AIC
Model 1 (face-to-face)	27	4,364.56	.04	.09 (.09–.10)	.94	4,400.56	N/A
Model 2^b^ (face-to-face)	26	3,383.53	.04	.08 (.08–.09)	.95	3,421.53	979.03
Model 3^c^ (face-to-face)	25	2,499.56	.03	.07 (.07–.08)	.97	2,539.56	881.97
Model 1 (telephone)	27	972.93	.04	.08 (.08–.09)	.92	1,008.93	N/A
Model 2^b^ (telephone)	26	854.18	.04	.08 (.08–.08)	.93	892.18	116.75
Model 3^c^ (telephone)	25	603.39	.04	.07 (.06–.07)	.95	643.39	248.78

*Note*. SRMR = standardized root mean square residual; RMSEA = root mea n square error of approximation; CFI = Comparative Fit Index; AIC = Akaike information criterion; PHQ-9 = Patient Health Questionnaire.

a All chi-square values are significant at *p* <. 001.

b In model 2, error variances of items 7 and 8 were correlated.

c In model 3, error variances of items 3 and 4 were correlated, as well as items 7 and 8.

The telephone data emerged as multivariate normal (Mardia's coefficient = 3.00), despite items exceeding the critical value for Mahalanobis distance. Fit indices for a unidimensional PHQ-9 were acceptable; with modification indices suggested the possible utility of correlating the error variances of items 7 and 8, and 3 and 4, as in the face-to-face data. When items 7 and 8's error variances were correlated, fit indices were better. Fit indices further improved when items 3 and 4's error variances were associated. The above models' fit indices also are in Table 2.

### Confirmatory factor analyses of the GAD-7

Face-to-face data emerged as multivariate non-normal (Mardia's coefficient = 11.14), with several variables exceeding the critical value for Mahalanobis distance (i.e., 24.32 for seven dependent variables). Using ML estimation, fit indices for a unidimensional GAD-7 were adequate. The biggest modification index suggested redundancy between items 4 and 5. This makes sense because these items represent restlessness and inability to relax. Their error variances were correlated, with improved fit indices emerging. The second largest modification index suggested that items 5 and 6 might have correlated errors. These items reflect irritability and restlessness, and hence correlated errors in this case make sense. Introducing these correlated errors resulted in improvements in fit indices. Table 3 displays the fit indices for these models.

**Table 3:** Confirmatory factor analysis for GAD-7 by mode of administration

Mode of administration	*df*	Chi-square^a^	SRMR	RMSEA (90% CI)	CFI	AIC	Delta AIC
Model 1 (face-to-face)	14	3,828.94	.04	.12 (.12–.12)	.94	3,856.94	N/A
Model 2^b^ (face-to-face)	13	2,504.89	.04	.10 (.10–.11)	.96	2,523.89	1,322.05
Model 3^c^ (face-to-face)	12	1,795.10	.03	.09 (.09–.09)	.97	1,827.10	707.80
Model 1 (telephone)	14	492.51	.04	.08 (.08–.09)	.96	520.51	N/A
Model 2^b^ (telephone)	13	290.21	.03	.07 (.06–.07)	.97	320.21	200.30
Model 3^c^ (telephone)	12	184.55	.02	.05 (.05–.06)	.98	216.55	103.67

*Note*. SRMR = standardized root mean square residual; RMSEA = root mean square error of approximation; CFI = Comparative Fit Index; AIC = Akaike information criterion; GAD-7 = Generalized Anxiety Disorder Scale.

a All chi-square values are significant at *p* <. 001.

b In model 2, error variances of items 4 and 5 were correlated.

c In model 3, error variances of items 5 and 6 were correlated, as well as items 4 and 5.

Telephone data also emerged as multivariate non-normal (Mardia's coefficient = 6.06) with some variables higher than the critical value for Mahalanobis distance. Using ML estimation, fit indices were satisfactory. Modification indices again highlighted shared variance among items 4 and 5, and 5 and 6. When the error variances of items 4 and 5 were associated, fit indices improved. Item 5's and 6's error variances then were correlated resulting, again, in better fit indices. These models fit indices are in Table 3.

### Factor loading invariance of the PHQ-9

The final model for the PHQ-9 was the same for face-to-face and telephone data, with error covariances between items 3 and 4, and 7 and 8. Therefore, it served as the baseline model to which the constrained model (with constraints on all parameters across modes of data collection) was compared. ML estimation determined the fit indices of the baseline model and the constrained model (Table 4). Comparing the fit of the constrained model with the baseline revealed a statistically significant difference in chi-square values, Δχ^2^(11) = 165.48, *p* <. 001, but a ΔCFI of −.002, suggesting the equivalence of factor loadings.

**Table 4:** Factorial loading invariance of the PHQ-9 and GAD-7

Measure and model	*df*	Chi-square^a^	SRMR	RMSEA (90% CI)	CFI	AIC	Delta AIC
PHQ-9 (baseline)	50	3,102.95	.03	.05 (.05–.05)	.963	3,182.95	N/A
PHQ-9 (constrained)	61	3,268.43	.04	.05 (.05–.05)	.961	3,326.43	−143.48
GAD-7 (baseline)	24	1,979.64	.03	.06 (.06–.06)	.975	2,043.64	N/A
GAD-7 (constrained)	33	2,144.88	.04	.05 (.05–.05)	.973	2,190.88	−147.24

*Note*. SRMR = standardized root mean square residual; RMSEA = root mean square error of approximation; CFI = Comparative Fit Index; AIC = Akaike information criterion; GAD-7 = Generalized Anxiety Disorder Scale; PHQ-9 = Patient Health Questionnaire.

a All chi-square values are significant at *p* <. 001.

### Factor loading invariance of the GAD-7

As the final model for the face-to-face and telephone data contained error covariances between items 4 and 5, and 5 and 6, it served as the baseline. Fit indices for it and the constrained model are in Table 4. Comparisons between the baseline and constrained models showed a statistically significant difference in chi-square values, Δχ^2^(9) = 165.24, *p* <. 001, but a ΔCFI of −.002. Thus, the model appeared invariant across the data collection modes.

### Invariance of the PHQ-9's latent mean structures

Error covariances, between items 3 and 4, and 7 and 8, were included and then the models (face-to-face vs. telephone) were compared. The critical ratio value for the latent mean estimate for the face-to-face data, which represented latent mean differences between the two groups, was −1.94, *p* =. 07. Therefore, there was no statistically significant difference in latent means as a function of data collection methodology. Acceptable fit indices increased confidence in the interpretation of estimates associated with the current solution: χ^2^(66) = 3,439.94, *p* <. 001; SRMR =. 03; RMSEA =. 05 (90% CI:. 05–.05); CFI =. 96; and AIC = 3,323.95.

### Invariance of the GAD-7's latent mean structures

Before comparing the models, error covariances, between items 4 and 5, and 5 and 6, were specified. The critical ratio value for the latent mean estimate for face-to-face data was −7.57, *p* <. 001, indicating a statistically significant difference in latent means between the modes of data collection (i.e., the latent mean for face-to-face data was less than that for telephone data). Adequate fit indices supported this interpretation: χ^2^(36) = 2,066.92, *p* <. 001; SRMR =. 03; RMSEA =. 05 (90% CI:. 05–.05); CFI =. 97; and AIC = 2,134.91.

### Tests of convergent validity

As hypothesized, positive correlations emerged between scores on the PHQ-9 and GAD-7. For face-to-face data, *r*(18,500) =. 74, *p* <. 001. A positive correlation among these variables also emerged in the telephone data, *r*(5,083) =. 67, *p* <. 001.

## Discussion

This study psychometrically evaluated the PHQ-9 and GAD-7 using first-contact data from patients accessing psychological services in one of the sites of the English IAPT programme. The questionnaires demonstrated strong psychometric properties with data collected face-to-face or via telephone. Favourable Cronbach's alphas indicated that the PHQ-9 and GAD-7 yielded internally consistent scores and, in support of previous psychometric work on the PHQ-9 (e.g., Cameron *et al*., 2008) and GAD-7 (e.g., Dear *et al*., 2011), confirmatory factor analyses suggested the questionnaires' one-factor models adequately matched observed face-to-face and telephone data. However, as modification indices suggested shared content among some PHQ-9 and GAD-7 items, and fit indices improved when relevant items' error terms were correlated, the PHQ-9 and GAD-7 items may possess overlaps in item content. Given this, clinicians may consider the use of abbreviated screeners for depression and anxiety, such as Kroenke, Spitzer, Williams, and Löwe (2009) brief screening scale for anxiety and depression, the Patient Health Questionnaire for Depression and Anxiety (PHQ-4). However, such consideration should be informed by the questionnaires' respective psychometric and pragmatic characteristics, such as sensitivity to change in response to treatment (Kroenke, Spitzer, Williams, & Löwe, 2010).

Factor loading invariance analyses suggested that items on the PHQ-9 and GAD-7 were invariant, equivalently operating across data collection modes. That is, PHQ-9 and GAD-7 items related to their respective latent variables in a comparable manner, regardless of data collection method. However, only the PHQ-9's latent mean structures were invariant, with patients answering the GAD-7 by telephone reporting greater anxiety. It is possible that socially anxious people are less likely to answer questions face-to-face (Erwin, Turk, Heimberg, Fresco, & Hantula, 2004) and, therefore, may feel more comfortable sharing their thoughts by telephone. Clinicians may hold this in mind when interpreting patients' responses to treatment using GAD-7 scores that were collected differently (e.g., first by telephone and then face-to-face). Future research may randomly assign patients to either complete the GAD-7 by telephone or face-to-face. This may help explain whether different latent means are attributable to the contrasting natures of these data collection methods or if people with more anxiety are more likely to provide data by telephone.

Finally, the associations between the PHQ-9 and GAD-7 supported the questionnaires' convergent validity. Correlations were positive and large (Hojat & Xu, 2004) suggesting that, on average, patients who presented to the IAPT service tended to report comorbid symptoms of depression and anxiety. This common type of comorbidity may predict negative health outcomes such as increased risk of suicide and treatment non-adherence (Hirschfeld, 2001), and research has found that overlap between depression, anxiety, and somatisation accounted for greater variance in functional impairment than the unique contributions of each problem alone (Löwe, Spitzer, *et al*., 2008). This underlines the importance of Roth and Pilling's (2008) meta-competences that assist clinicians' management of complex comorbidity (Rector, 2012).

The current research improved understanding of the PHQ-9 and GAD-7's psychometric properties; however, more psychometric research is recommended. Researchers should investigate the factorial invariance of the PHQ-9 and GAD-7 using data from other IAPT sites. If appropriate, such research may include other forms of data collection such as email, the use of which has been suggested by IAPT (Department of Health, 2011). Research has attested to the psychometric properties of the PHQ-9 (Titov *et al*., 2011) and GAD-7 (Donker, van Straten, Marks, & Cuijpers, 2011) among Internet samples. However, measurement invariance research on measures of social anxiety has suggested that the constructs manifested differently across the administration modalities of online at home and paper-and-pencil in a laboratory setting (Hirai, Vernon, Clum, & Skidmore, 2011).

Future research may also address limitations of this study. Other outcome measures (e.g., IAPT phobia scales) were not imported from PC-MIS, precluding potentially informative tests of validity; and data collectors were unidentifiable, disallowing analyses stratified by profession. This may be important because low-intensity psychological wellbeing practitioners might be more likely to collect data via telephone than high-intensity cognitive behavioural therapy workers.

### Conclusion

Although further psychometric work is warranted, the current research supported the internal consistency and convergent validity of the PHQ-9 and GAD-7; suggested the appropriateness of unidimensional factor structures for these questionnaires; deemed that both questionnaires' factor loadings were invariant across modes of data collection; and showed that only the PHQ-9's latent mean structures were equivalent across groups. This means that, based on the current research findings, clinicians may be confident collecting PHQ-9 data by telephone and face-to-face and, then, comparing such data. For example, an IAPT clinician may gauge a patient's progress in therapy by comparing their end-of-therapy PHQ-9 to their start-of-therapy PHQ-9 scores, even if these data were collected by telephone at the start and face-to-face at the end, or *vice versa*. However, clinicians are recommended to reflect on how the GAD-7's latent means differed if they want to determine clinical effectiveness using telephone and face-to-face GAD-7 data.

## Footnotes

1 Analyses were carried out with bootstrapping and ML estimation, producing very similar results. Therefore, ML estimation's findings were reported throughout.
